# Metastatic Translocated Renal Cell Carcinoma in a Kidney Transplant Patient - a Case Report and Review of the Literature

**DOI:** 10.1177/10668969231185070

**Published:** 2023-07-06

**Authors:** Johannes Kläger, Manuela Schmidinger, André Oszwald, Gabriel Wasinger, Harun Fajkovic, Eva Compérat

**Affiliations:** 1 Department of Pathology, 27271 Medical University of Vienna, Wien, Austria; 2 Department of Urology, 27271 Medical University of Vienna, Wien, Austria

**Keywords:** TFEB-altered renal cell carcinoma, TFEB translocation, RCC metastasis in kidney transplant, urological pathology, metastatic TFEB translocation RCC

## Abstract

*TFEB*-altered renal cell carcinomas are rare tumours. Here, we report the exceptional case of such a tumour in the setting of solid organ transplantation and with already metastatic disease at the time of diagnosis. The primary tumour occurred in the native kidney and only focally showed biphasic morphology whereas the metastasis, among others to the transplant kidney, showed nonspecific, albeit different morphology, but both had consistent *TFEB* translocation. Treatment with the immune checkpoint inhibitor pembrolizumab together with the multi-kinase inhibitor lenvatinib achieved partial response 14 months after diagnosis.

## Introduction

In 2022, the new edition of the WHO Classification of Tumours series of “Urinary and male genital tumours”^1^ was released. A major novelty is the introduction of a new category of molecularly-defined renal tumour entities to identify morphologically heterogeneous tumours with characteristic recurrent genetic alterations. One of the entities included in this category is the *TFEB*-altered renal cell carcinoma, initially included in the 2016 WHO classification as a member of the microphthalmia (MiT/TFE) transcription factor family translocation carcinomas.

These rare tumours are defined by a translocation of *transcription factor EB* (*TFEB*) on chromosome 6 and most often, but not exclusively, chromosome 11 (t(6;11)(p21;q12) translocation), or by amplification and consequent overexpression of the *TFEB* gene.^2^ In general, *TFEB*-translocated tumours have a favorable prognosis, but rare metastatic tumours have been described. In contrast, *TFEB*-amplified tumours with or without concomitant *TFEB* translocation show a more aggressive behaviour with frequent metastasis, emphasizing the necessity to distinguish these tumours.^3-5^

Here we report a metastatic *TFEB*-translocated, but not amplified, renal cell carcinoma in a kidney transplant patient who was consequently treated with immune therapy and tyrosine kinase inhibitors and provide a review of the current literature.

## Material and Methods

The patient gave consent to process their data. Histologic specimens were formalin-fixed and paraffin-embedded according to routine protocols. For histological evaluation, 3 µm sections were used for histochemistry [hematoxylin and eosin (H&E), periodic acid–Schiff (PAS), acid fuchsin orange G (AFOG), methenamine-silver] and immunohistochemistry [TFE3; Cell Marque (354R), Rocklin, California 95677, USA], keratin 7 [KRT7; Agilent Dako Omnis (GA61961-2), Santa Clara, CA 95051, USA], carbonic anhydrase 9 [CA9; Abcam (AC-0137RUOC), Cambridge, UK], paired box 8 [PAX8; Cell Marque (363M), Rocklin, California 95677, USA], melan A [Ventana/Roche (05278350001), 68305 Mannheim, Germany], anti-melanosome [HMB45; Agilent Dako (M063429-2), Santa Clara, CA 95051, USA], pan-keratin AE1/AE3 [Agilent Dako Omnis (GA05361-2), Santa Clara, CA 95051, USA], applying standard methodology.

Detection of *TFEB* translocation was performed by fluorescent in-situ hybridization (FISH) using 5 µm sections and a break-apart probe [EmpireGenomics (TFEBBA-20-ORGR), Williamsville, New York 14221, USA] according to the manufacturer's protocol. For evaluation of sex chromosome status, centromere probes for X and Y chromosomes [Vysis/Abbott (X: 32-112023, Y: 05J08-025), Abbott Park, Illinois, USA] were used, with green fluorophores for X- and red fluorophores for Y-centromer, according to the manufacturer's protocol.

## Results

The patient was a 62-year-old woman with renal failure for greater than 30 years due to post-streptococcal glomerulonephritis, with history of four consequent renal transplants. The most recent transplantation was in the late 2000s. Another known medical condition of potential importance for kidney tumourigenesis was controlled hypertension.

**Figure 1.**
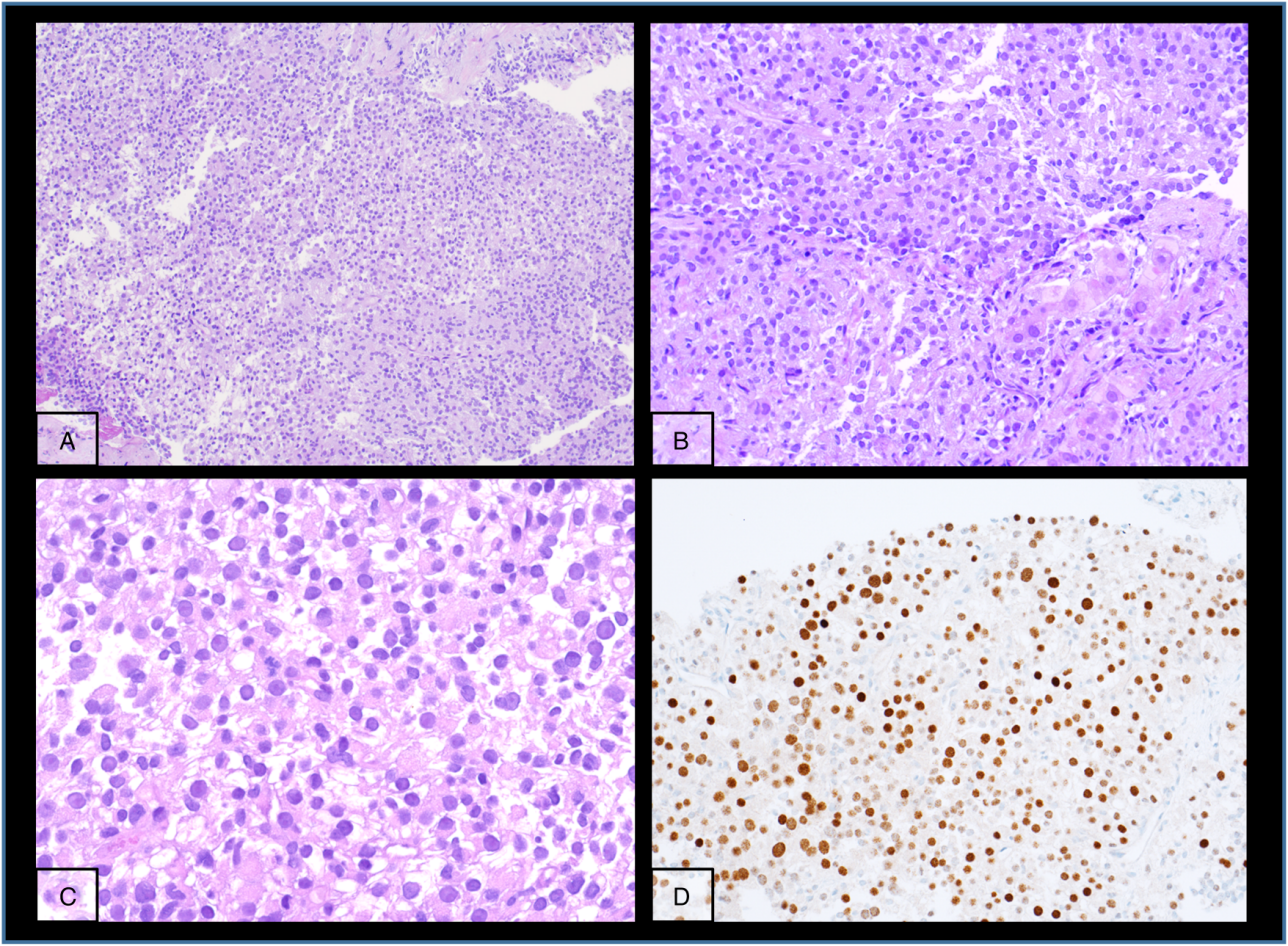
Metastatic lesion in the liver with nonspecific morphology but PAX8 positivity.

**Figure 2.**
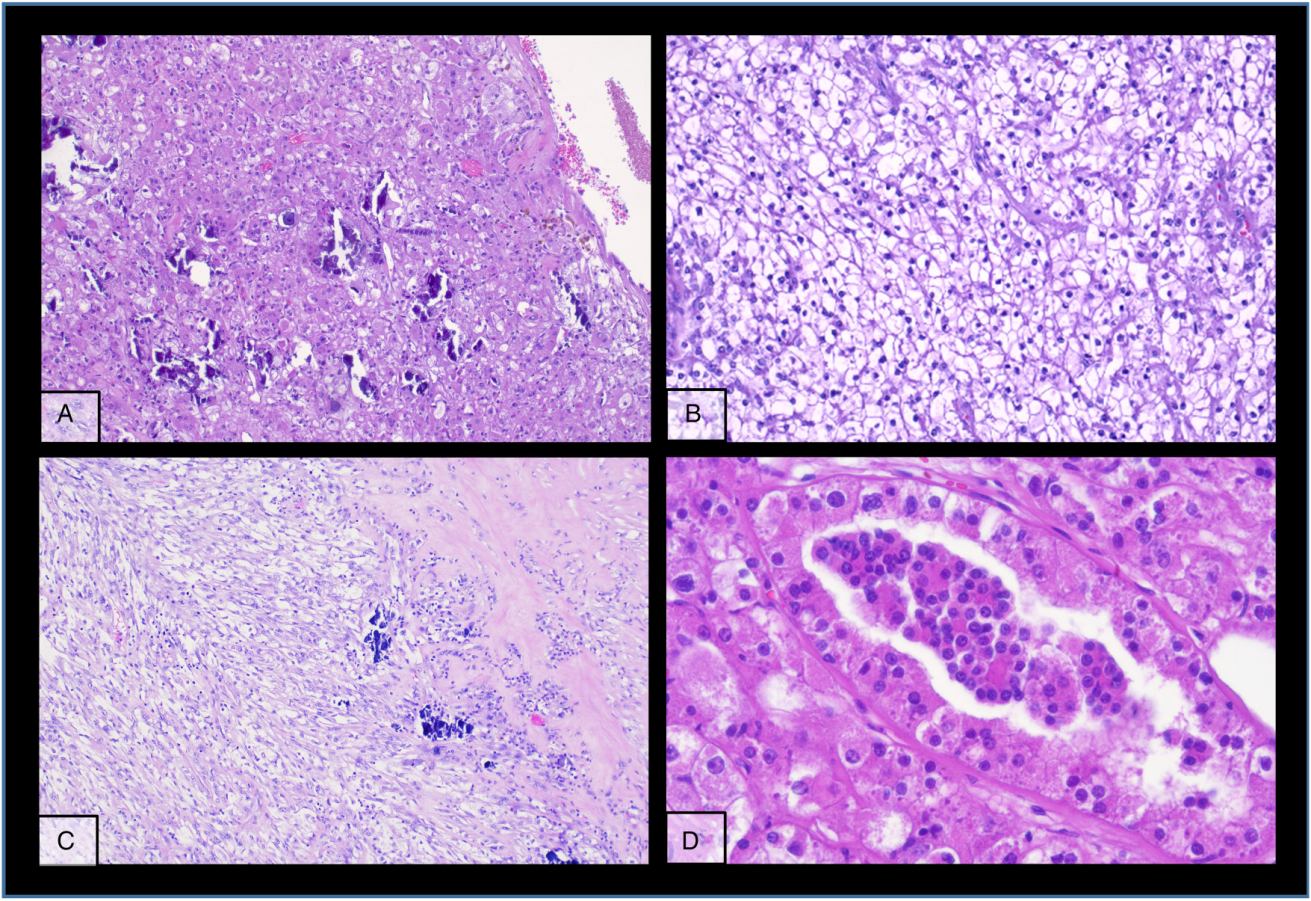
Different typical morphologic patterns of the primary tumour in the native kidney (A-D).

**Figure 3.**
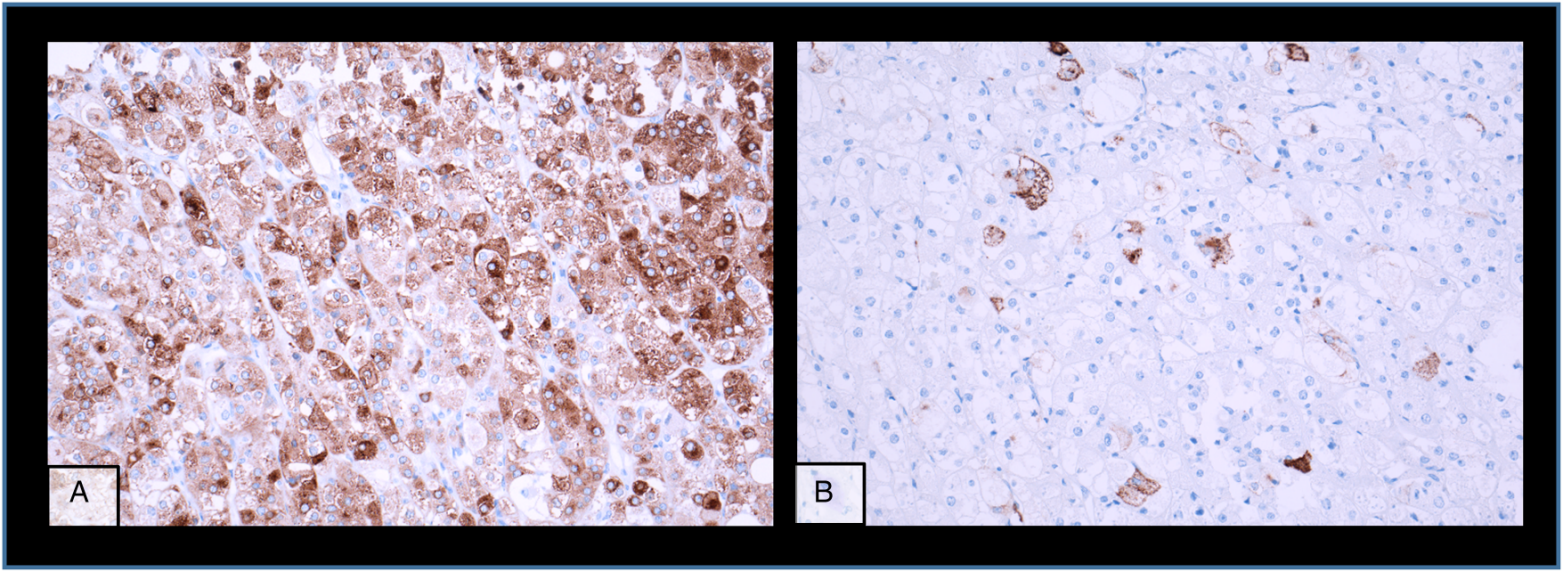
Immunohistochemistry staining of the primary kidney tumour with (A) Melan A and (B) HMB54.

**Figure 4.**
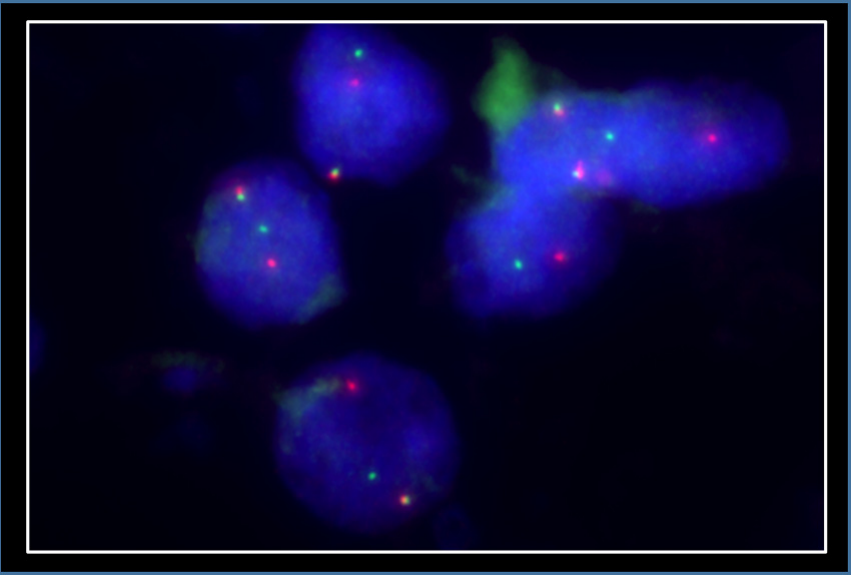
FISH showing TFEB translocation in the primary kidney tumour.

The kidney tumour was incidentally discovered in a computed tomography (CT)-scan for re-evaluation for another kidney transplantation due to progressive graft deterioration of unknown cause. A lesion of 11 cm diameter in the left native kidney and a hepatic nodule of 3 cm diameter were detected. Histologic evaluation of the hepatic nodule showed a neoplastic lesion with a nonspecific sheet-like growth pattern, low-grade polymorphism, and components with clear cell and eosinophilic morphology (Figure 1A-C). By immunohistochemistry, the neoplastic cells showed nuclear expression of PAX8 (Figure 1D), confirming renal origin, so nephrectomy was performed. The histological workup of the native left kidney showed a solid and partly cystic neoplasm with large areas of necrosis, hemorrhage and multiple psammoma bodies. The cells had a mixed morphology (clear cell and oncocytic) with focal areas with fibrosis and spindle-shaped cells (Figure 2A-C). We found only a single small focus with biphasic morphology typical for *TFEB*-altered tumours (Figure 2D). Immunohistochemistry for melan A showed strong diffuse tumour cell positivity, HMB45 was mainly negative with only single cell positivity (Figure 3). TFE3, CA9 and KRT7 were negative. FISH analysis revealed a *TFEB* translocation, but no amplification (Figure 4, split signal in 72% of cells), rendering the final diagnosis of a t(6;11) translocation renal cell carcinoma, pT3a pN0 pM1 (UICC eighth Ed).

**Figure 5.**
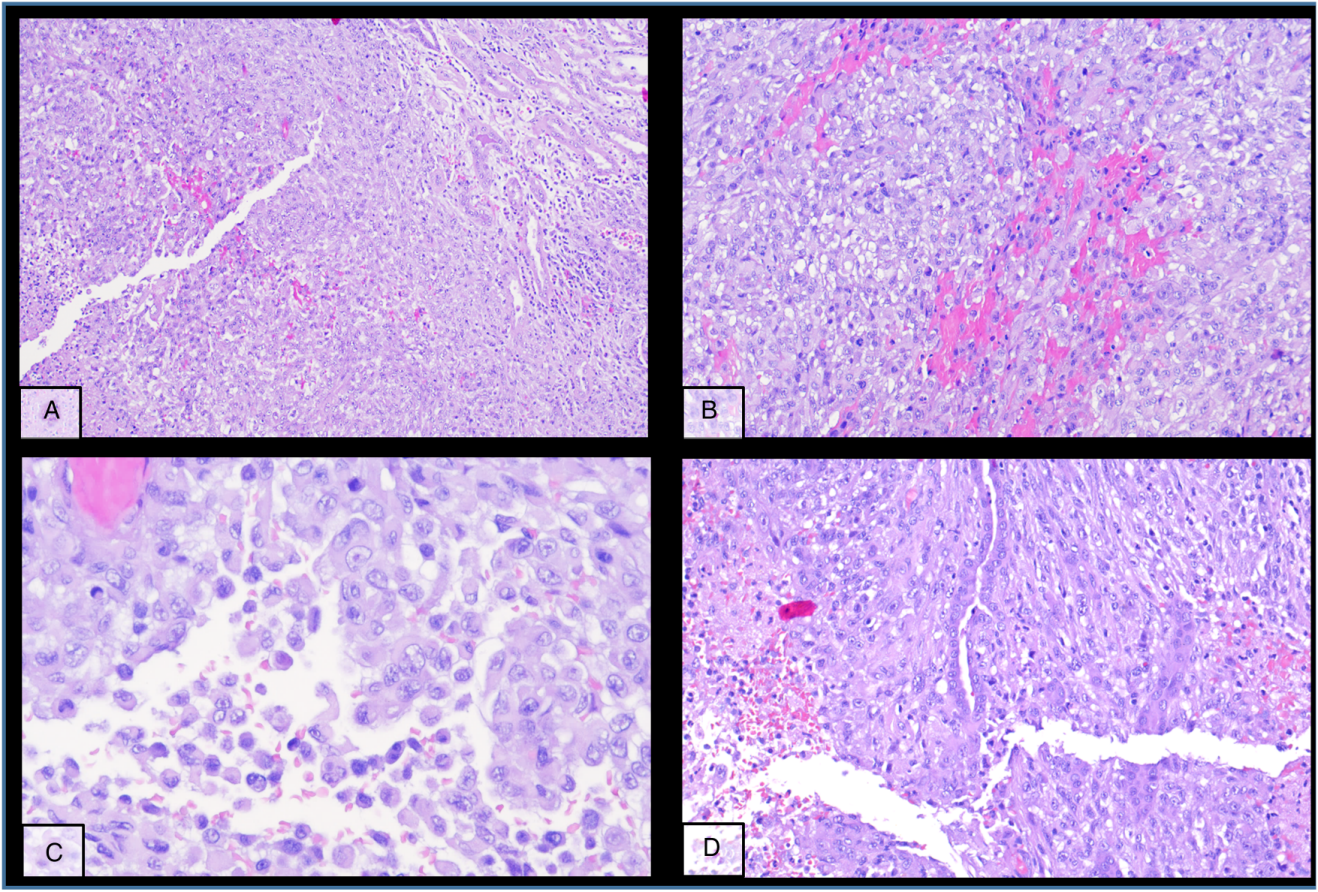
Monomorphic pattern of kidney metastasis (A-C) with sometimes incorporated pre-existing structures (D).

**Figure 6.**
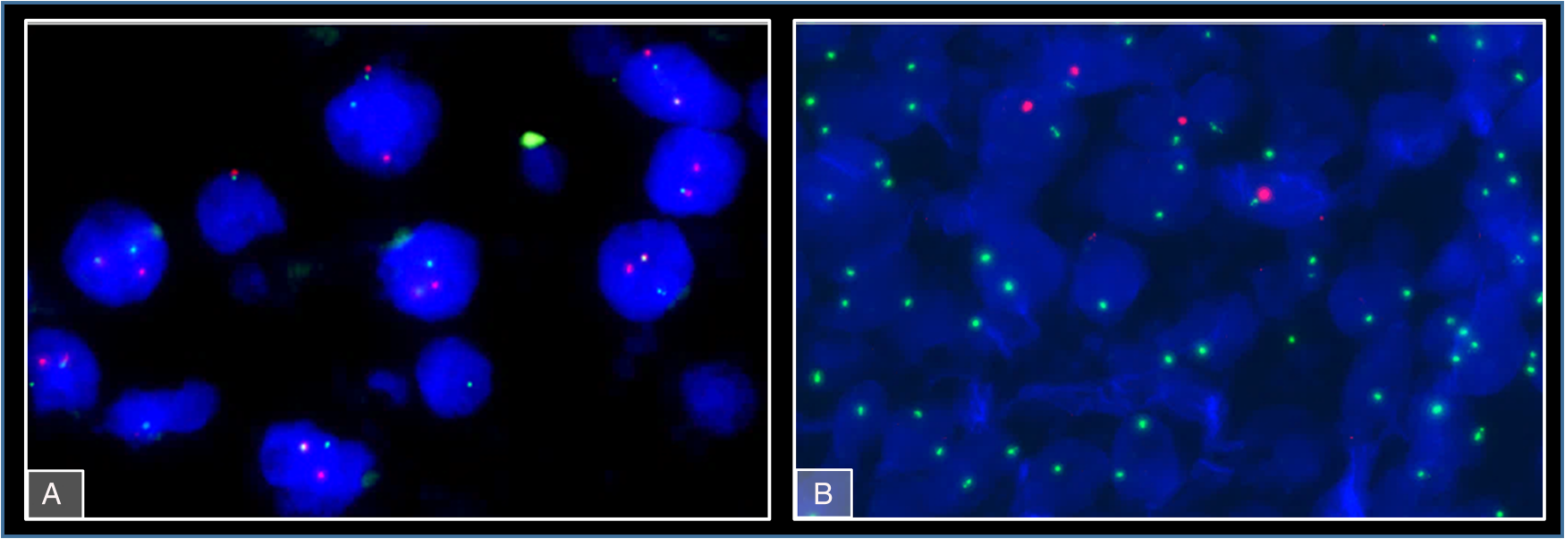
FISH showing TFEB translocation (A) and XX genotype (B) in metastatic tumour cells in the kidney allograft.

At that time, no further metastatic lesions were detected, and therefore the hepatic lesion was treated with radio frequency ablation. Four months after diagnosis, the patient's condition deteriorated and a CT-scan showed disease progression with new metastases to the liver, lung, and mediastinal lymph nodes, urging the need for systemic therapy. Immune checkpoint inhibitor (ICI) combinations are currently considered the standard of care in clear cell renal cell carcinoma. No standard of care exists for *TFEB*-altered renal cell carcinoma, however, guidelines recommend a treatment pattern similar to that of clear cell renal cell carcinoma for all non-papillary renal cell carcinomas. After discussion with the patient, the patient's nephrologist and oncologist, the decision was made to first remove the kidney transplant and subsequently be able to initiate treatment with the ICI pembrolizumab (Keytruda®, Merck/MSD, Rahway, NJ USA) combined with the *vascular endothelial growth factor* (*VEGF*)-inhibitor axitinib (Inlyta®, Pfizer, New York, NY, USA). At that time, the kidney graft had experienced slowly progressive functional loss, but there was no suspicion of metastasis. Surprisingly the pathological work up of the kidney transplant showed an uneven surface with multiple parenchymal nodules on cross-section. Microscopically, these nodules consisted of moderately circumscribed, non-encapsulated lesions of epithelioid- to spindle-shaped cells and central necrosis in the larger lesions. The cells had vacuolated to eosinophilic cytoplasm and nuclei of variable sizes with vesicular chromatin and prominent nucleoli (Figure 5A-D). In the periphery, preexisting structures were occasionally incorporated by the tumour (Figure 5D). The tumor cells were immunoreactive for pan-keratin (AE1/AE3) and PAX8, but no reactivity with KRT7, CA9 and TFE3. FISH-analysis detected a *TFEB* translocation (Figure 6A, split signal in 52% of cells), while additionally, centromere probes for chromosomes X and Y showed a single chromosome X in the neoplastic cells but both chromosomes X and Y in preexisting renal tissue. Since the patient was female and the kidney donor was male, this pattern of sex chromosomes confirms that the nodules in the kidney graft metastasized from the tumor in the native kidney (Figure 6A). The non-neoplastic parenchyma showed immune complex-glomerulonephritis but no signs of rejection.

Upon systemic treatment initiation, our patient showed partial response in the first follow-up CT scan 2 months later but progressive metastatic disease another 2 months later. Therefore, treatment regimen was changed to pembrolizumab combined with lenvatinib (Lenvima®, Eisai, Tokyo, Japan) which again led to partial response with stable disease 12 months follow up, still ongoing.

## Discussion

In general, transplant recipients have an increased risk of malignancies including a 6- to 15-fold increased risk of different subtypes of renal cell carcinomas.^6-8^ Most of the tumours occur in the remaining native kidney and only about 10%-20% originate from the kidney transplant.^7,9^ Despite the close association between acquired cystic disease-associated renal cell carcinomas and end-stage renal disease, the most common renal cell carcinoma subtypes in transplant recipients are clear cell and papillary renal cell carcinomas.^7,9^ Most of the renal cell carcinomas arising in kidney transplant recipients are limited to the kidney irrespective whether they arise in the native or the transplanted kidney.^9-11^ However, some examples with distant metastasis are reported but none of them into the renal allograft.^9,11-16^ Also, tumours of other origin than kidney seem to rarely metastasize into the allograft in kidney transplant recipients. We could only find one single report of a metastatic allograft lesion originating from a urothelial carcinoma of the recipient.^17^ Another possible risk factor for developing malignancies is chemotherapy, a fact well recognized for acute leukemia but also other tumor risks might increase.^18^ Argani et al described six patients out of a cohort of 39 children and adolescents/young adults developing MiT/TFE translocation renal cell carcinoma after exposure to cytotoxic chemotherapy for malignant or autoimmune diseases, suggesting an association of some chemotherapy substances with occurrence of translocation renal cell carcinoma in young patients.^19^ Our patient suffered from poststreptococcal glomerulonephritis and progressed to end-stage kidney disease and dialysis within 2 years in her mid-twenties. Unfortunately, there are no records about the medication during this time where she might have received such medication. However, potential treatment interval was less than two years, whereas in aforementioned cohort it was six years median (IQR: 4,5-7,5a) for those who developed MiT/TFE translocation renal cell carcinoma.^19^ Yet, one patient`s treatment interval was only two years also. Here, we report an example of *TFEB*-translocated renal cell carcinoma in a kidney transplant patient with the unusual event of distant metastasis in multiple organs, including the kidney transplant, which, to our knowledge, has not been reported before.

*TFEB*-translocated tumours are a molecularly-defined and globally rare entity with only about 100 reported examples, making it difficult to generalize conclusions at this time.^20^ They are characterized by a gene fusion involving the *transcription factor EB* (*TFEB*), most commonly with *MALAT1.*^21,22^ In the WHO Classification of Tumours series of “Urinary and male genital tumours”, it is described that these tumours may have a quite exceptional morphologic biphasic pattern of larger epithelioid and smaller cells clustered around extracellular hyaline material, however, this pattern is not mandatory. Indeed, a broad range of morphology, including, among others, clear cell, papillary, oncocytic or spindle cell-like shape, has been observed.^23^ Moreover, there is no specific immunohistochemistry, although remarkably, most tumours stain positive with Melan A or Cathepsin K. Ultimately, FISH or other molecular evidence of *TFEB* translocation is required for diagnosis.^3,5,23,24^

In our patient, the primary tumor only focally showed a small area with distinctive biphasic morphology. Such small areas might easily be undersampled at specimen grossing and or missed at microscopic evaluation. The metastatic nodules also had no distinctive biphasic morphology, but both metastasis and primary tumour samples showed *TFEB* translocation without amplification in FISH analysis. Notably, the morphology in the transplant kidney metastasis differed considerably from the primary tumour, whereas the metastatic liver nodules were more similar.

In most cases, *TFEB* translocated renal cell carcinomas behave indolently but there are some reports of metastatic disease and cancer-related deaths.^3,25^ Most patients present with an early tumor stage, where resection is sufficient, and no systemic therapy is applied. In our patient, however, the patient was incidentally diagnosed with limited metastatic disease during evaluation for kidney re-transplantation. However, 4 months after diagnosis, the patient exhibited progressive and widespread metastatic disease. In the literature, Peckova et al. and Caliò et al. described five tumours with *TFEB* t(6;11) with recurrence or metastasis.^3,4,26^ Morphologically, most patients had large primary tumour size, occurred in older patients and often showed grossly visible necrosis.^3,4^ Importantly, these aggressive tumours also had *TFEB* amplification, a genetic alteration associated with more aggressive behaviour^27,28^ emphasizing the necessity to distinguish them from purely *TFEB* translocated tumors. In a retrospective analysis by Xia et al of 25 tumors with *TFEB* translocation without amplification, 2 showed progressive disease and multifocal or grossly visible necrosis was identified as the only morphologic parameter for more aggressive behaviour.^22^ Our patient´s primary tumour size was 11 cm, with 20% tumour necrosis detected microscopically. She received immunosuppressive drugs due to her kidney transplantation. This circumstance might also contribute to a more deleterious disease course due to impaired immunosurveillance.^29^ It is unclear which of the currently available drugs should be administered for systemic therapy, but several small studies indicate potential benefit of immune checkpoint inhibitors.^30,31^ A gene expression study by Bakouny et al. showed high activity in the *NF-E2-related factor 2* (*NRF2*) pathway in translocated renal cell carcinomas, a finding which is associated with reduced response to targeted therapy in vitro and with reduced progression-free survival in therapy regimens without immune checkpoint inhibitors in vivo.^21^ Additionally, Zhang et al. showed that *TFEB* can also upregulate *PD-L1* expression and might lead to immune evasion.^32^ Our patient began treatment with pembrolizumab and axitinib, which resulted in partial remission lasting for 4 months. Upon disease progression, the patient received a second-line treatment with pembrolizumab and lenvatinib, which achieved partial remission still lasting 12 months after initiation.

## Conclusion

*TFEB*-altered tumours, consisting of *TFEB*-translocated and amplified tumours, differing in their biological behaviour, are rare entities and even more so in the setting of solid organ transplantation. Here, we report a rare example of metastatic *TFEB*-translocated tumour in a kidney transplant patient. For diagnosis, demonstration of *TFEB* alteration using FISH or sequencing techniques is mandatory, as these tumours exhibit a broad morphologic range. Options for systemic treatment are still a matter of debate but immune checkpoint inhibitors and tyrosine kinase inhibitors led to partial remission in our patient.
